# Dr. Nisbet, on Cancerous Diseases

**Published:** 1802-10-01

**Authors:** W. Nisbet

**Affiliations:** London Street, Fitzroy Square


### To Dr. BATTY,

Dear Sir,

In your next Number I fhall trouble you with two cafes of Uterine Cancers fuccefsfully treated; in the mean time, your infertion of the following letter on a fubject of the firft importance in Medicine, and which fhall be continued at fome length in the future Numbers of your ufeful publication, will allow the opinions I entertain of it to be very generally known to the Gentlemen of the Profeffion, will enable them to correct miftakes miſtakes in my views of it, and to bring the principles of treatment, held out to the teſt of experience, by means of their own practice. I am, &c.

*London Street, Fitzroy Square,* W. NISBET.

*Sept.* 23, 1802.

---

LETTER TO SIR W. FARQUHAR, BART. ON THE PRINCIPLES TO BE ADOPTED IN THE TREATMENT OF CANCEROUS DISEASES.

SIR,

WHEN I had laſt the pleaſure of ſeeing you, from the particular line of practice I had adopted, our converſation was naturally directed to the ſubject of Cancer. You then told me, with a frankneſs of character to which I gave every credit, that you did not believe a Cancer could be cured; and I, with equal candour, replied,—I did not wiſh you to believe any thing of which you had not complete evidence. It is from this aſſertion, on my part, and to ſupport the opinion I then, and have uniformly delivered, that I now addreſs you in the preſent obſervations, which I flatter myſelf will be found to meet approbation, and to open a new field of practice in this diſeaſe. I addreſs them more eſpecially to you, as having ſo ſtrongly declared your ſcepticiſm on the ſubject, and alſo from your rank in Medicine, as you ſtand in that prominent ſituation which muſt influence the opinion of inferior practitioners.

In a late publication on the two diſeaſes of Scrofula and Cancer, I took a retroſpective view of what had been done by authors previous to the preſent period, in order to elucidate their nature and treatment, without detailing any particular opinions of my own,

In an Appendix to a ſecond edition of that work, containing a letter addreſſed to Profeſſor Gregory, in conſequence of ſome correſpondence on the ſubject with which you were acquainted, I entered into ſome detail of my own views on theſe diſeaſes; and it is my deſign to unfold more at large what increaſed experience, a detection of error, and more enlarged obſervations, have confirmed.

The three great diſcoveries which have materially influenced the practice of Phyſic, in modern times, are, the diſcovery of the Circulation of the Blood, the diſcovery of the Lymphatic Syſtem, and the Uſes of Reſpiration. The firſt of theſe has been ſufficiently appreciated by moſt writers, and the knowledge of it applied, with much ſucceſs, to the treatment of acute diſeaſes. The ſecond of theſe is daily gaining gaining more importance, and the application of it is making, with much advantage, to the cure of moſt chronic affections. Both theſe diſcoveries, however, may be conſidered as imperfect, till the funǈton of Reſpiration was chemically examined, and the changes induced by it on the animal ſyſtem detected.

In order to be fully ſatisfied of this important truth, it will be neceſſary, as a preliminary ſtep; firſt, to take ſome view of this diſcovery; ſecondly, to conſider its influence on the Glandular or Lymphatic Syſtem in particular; and having done this, laſtly, to apply the facts deduced from this foundation to the explanation and treatment of the preſent diſeaſe.

The extent of the changes induced by Reſpiration are, firſt, to convey to the blood, and of courſe, in a ſecondary way, to the ſolids, that principle which is the baſe of vitality, and which, under the name of Oxygen, is found eſſential to the ſupport of life. It is conveyed in a conſtant and regular manner by a principal organ, appropriated to this particular purpoſe, and the quantity of it taken into the ſyſtem by every inſpiration of this organ, is equal to a hundred and twelve cubic inches in the minute. For we find, that the lungs inſpire in this period fifteen times, and at each inſpiration they carry in thirty cubic inches of atmoſpherc air, while the proportion of vital air being only a fourth of this, the quantity we have ſtated will be found correct. Of this quantity alſo, one half is immediately diſcharged by the ſame action of the lungs, in combination with two other principles, known under the name of Carbon and Hydrogen, extricated from the body. The two firſt conſequences of the introduction of this principle into the ſyſtem, is every part receiving increaſed animation and action, which often takes place to ſuch a degree as to induce, from its exceſs, peculiar modifications of diſeaſe. Along with this increaſe of animation, an increaſe of temperature alſo enſues, and the animal heat we find communicated in proportion to the diſtribution of Oxygen through the body, or the frequency of inſpiration in the different claſſes of animals; which introducing, of courſe, a greater quantity of this matter, occaſions the various proceſſes of chemical combination between the ſolids and fluids to be oftener repeated; proceſſes, the great ſource of the extrication of heat. The third effect of the introduction of this principle into the ſyſtem, and one materially connected with our views of the preſent diſeaſe, is the giving to the fluid that perfect ſtate which renders it fit for the renewal of the different ſolid parts of the ſyſtem, and for repairing that waſte in their ſtructure which neceſſarily accompanies the exerc ſe of their functions, and occaſionally is a conſequence of their injury from external violence or other cauſes. This ſtate conſtitutes the formation formation of what is termed Fibrina, the conftituent part of all the folids of the body; a part which neither exifts in the chyle nor lymph till the effects of refpiration are communicated to the fluids. On whatever peculiarity of combination this change depends, we find one circumftance always neceffary to its taking place; this is, the abftraction of two principles formerly noticed, Carbon and Hydrogen, in a certain degree, which principles conftitute the bafe of moft of the nourifhment the body receives. This abftraction, therefore, may be equally carried to excefs; and from this fource of too ftrong a difpofition to the formation of Fibrina, difeafes may arife, as well as from the caufe formerly noticed. This will be the more particularly apt to occur in the minute parts of the fyftem, where a natural tendency to obftruction prevails, and where the lymph, the part of the fluid moft concerned in the formation of Fibrina, and liable fooneft to affume this ftate, only circulates. In order to judge properly of this effect, it will be neceffary here to take fome view of that minute and extenfive divifion of the vafcular fyftem, termed Lymphatic; firft, as to its arrangement and diftribution; and, fecondly, as to its nature and properties.

The Lymphatic Syftem takes its origin every where from the furfaces of the body, external and internal, and the extremities of its fmall irritable veffels, open by what are called pores. Their figure is nearly cylindrical; they are plentifully filled with valves, in order to prevent the regrefs of their contents; and they terminate at certain places in glands which confift of the union and ramification of a number of their fingle tubes. The fluid they contain is entirely of a pellucid nature, from whatever fource it is derived. The extent of thefe veffels is much greater than any other feries in the body. Their number, in particular places, feems proportioned to the neceffity that there is for their ufe; but, on a general calculation, it appears from diffection, that for one artery we find two veins, and no lefs than four lymphatics. Hence the Lymphatic Syftem bears a proportion to the other veffels, as four to one; and hence, in the fame proportion, is it liable to prove the fource of difeafe. The valvular conftruction of the Lymphatics has been confidered as one of the leading diftinctions from the other veffels, a diftinction which has even given them name. Hence the peculiarity of their conftruction, neceffary, as it would appear, to the paffage of their fluids in animals which ufe the erect pofture, is one fource of their liability to difeafe, as permitting no retrograde motion when obftruction once occurs. In the courfe of their progrefs, the lymphatics terminate in fmall knots or clufters, termed from their fhape conglobate Glands. When they reach thefe glands they divide into a very great number of branches, which which are ſo minute, that it is difficult to force a penetrating injection through them. When ſuccefsful by this injection, it is found, that after dividing within the gland, the extremities of the veſſels open into cells which are natural to theſe glands, and in which their contents are depoſited. In tracing the lymphatics through their courſe, two ſets of them are conſpicuous, an external and an internal. The former accompanies the ſubcutaneous larger veſſels, the latter the arteries and deep-ſeated veins. It is the former that chiefly claims attention, as they are almoſt excluſively the ſeat of this diſeaſe. Hence Cancer is oftener met with in the breaſt, axilla, and jaw, where theſe glands are moſt numerous, than in any other parts of the body. From this ſtructure of the glands, the minute diviſion of their veſſels, the valvular conſtruction of theſe veſſels, and the numerous follicles or cells into which the glands divide, the tendency to obſtruction muſt, at all times, be great, and liable to take place on the ſlighteſt injury or derangement of their circulation. We can have no doubt that this ſtructure is eſſentially neceſſary to the different purpoſes of ſecretion; and as it is ſo, we find Nature endeavour to counteract the tendency to obſtruction and diſeaſe, by conſtantly embedding all theſe glands in a quantity of fat or adipoſe ſubſtance, as a guard for their protection from injury, to facilitate their circulation, and by its abſorption to ſerve other purpoſes to be afterwards noticed. Hence the fat is of the utmoſt conſequence to theſe glands, in continuing their folliculous veſſels in a healthy and permeable ſtate.

This circumſtance of the accumulation and changes on the ſituation of the fat in the progreſs of life is a ſubject of the firſt importance, as ſhewing the efforts of the ſyſtem to prevent its annihilation. In the earlier period of exiſtence, when the vital powers are ſtrong and active, Nature is chiefly anxious to preſerve the life and circulation of the exterior and ſmaller veſſels. The fat therefore, as the means of doing this, is chiefly accumulated on the external parts, and thence as it muſt, like the reſt of the body, ſuffer a perpetual ſeries of changes, it is reſumed into the circulation, and ſupplies that principle which the body requires in addition to the other ones received through the principal organs to the general circulation. As life advances this diſtribution of the fat becomes changed. It gradually leaves the external parts, and is drawn from the ſurface to the interior receſſes. Thus Nature forſaking, as it were, the more diſtant parts of the ſyſtem which are doomed to decay, and which ſhe is no longer able to ſupport, accumulates that principle which preſerved the exterior parts in health, on the internal viſcera, for the purpoſe of preſerving the larger veſſels, and more vital parts, as long as poſſible, from falling into the ſame ſtate,

ſtate. By this beautiful diſtribution the energies of life are longer maintained; nor are the changes on the quality of the fat leſs conſpicuous than its ſituation. The fat of the child is firm and inſulated, ſo that the abſorption of it may not take place in a degree beyond what is neceſſary for the wants of the ſyſtem. In manhood alſo it is diſtinguiſhed by conſiderable firmneſs, without much humidity or oilyneſs; but in age it is ſo remarkable for the change, and is of ſuch a fluid oily nature, that every anatomiſt has taken notice of it as one of the chief inconveniences that attends the diſſection and preparation of older ſubjects. On the ſame principle, that the fat ſerves to preſerve the vitality of the ſmaller veſſels in early life, the accumulation of this ſubſtance can be accounted for in all the animals of the colder regions. The leſſened temperature, which particularly attacks the circulation of the ſurface and minute parts, is thus counteracted; while in the warmer regions, from the oppoſite cauſe, and the more invigorated ſtate of ſurface, no accumulation of this ſubſtance takes place; on the contrary, rather a deficiency prevails. The compoſition of fat, when chemically examined, we find to conſiſt of five parts of Carbon and one of Hydrogen. Its accumulation by the ſmaller veſſels in diſtant parts, ſhews that theſe principles abound in the ſyſtem which occaſions this accumulation, and therefore when a change takes place, either they do not longer remain in the ſyſtem in the ſame quantity as before, or in the ſame proportion; or, if ſo, the veſſels are no longer ſuited to make the ſame extrication of them as before.

From this ſtatement, we are induced to conclude, that at the period when Cancer is apt to occur, an alteration in this fatty ſubſtance prevails. It acquires a morbid firmneſs or conſiſtence, and is no longer capable of conveying to the gland that utility it formerly diſplayed. At the ſame time, the veſſels themſelves begin to loſe that energy conſpicuous at an earlier period of life. The concurrence of theſe circumſtances, we contend, produce all the conſequences that enſue in Cancer, and that this diſeaſe conſiſts in an exceſſive or morbid diſpoſition to the formation of Fibrina, which, though general throughout the ſyſtem, is particularly diſplayed in thoſe parts where the ſtructure, nature of the circulation, and changes induced by period of life, favour it more ſtrongly than elſewhere. To render this clear and perſpicuous, we ſhall examine the diſſection of the parts in this diſeaſe, as far as theſe diſſections have been made by authors. All ſuch diſſections uniformly concur in the regularity of theſe morbid appearances.

1. The firſt is a thickening of ſubſtance in the parts.

2. The ſecond is an increaſed hardneſs, amounting often to a cartilaginous ſtate.

3. The

3. The third is the appearance of membranous ſepta, where no membrane was formerly conſpicuous; an appearance remarked by Dr. Baillie as ſtrongly characteriſtic of this malady.

From theſe facts there clearly appears an appoſition of ſolid matter to the part; and this appoſition naturally preſuppoſes, 1ſt. A morbid depoſition of the principles which go to frame it; and, 2d. An obſtruction in the parts to the removal of this depoſition when thus made.

With this knowledge of the actual morbid ſtate, we are naturally led to a nearer view of the principal ſymptoms which characteriſe the progreſs of the malady.

[ To be continued. ]

